# Nur77 prevents excessive osteoclastogenesis by inducing ubiquitin ligase Cbl-b to mediate NFATc1 self-limitation

**DOI:** 10.7554/eLife.07217

**Published:** 2015-07-14

**Authors:** Xiaoxiao Li, Wei Wei, HoangDinh Huynh, Hao Zuo, Xueqian Wang, Yihong Wan

**Affiliations:** 1 Department of Pharmacology, University of Texas Southwestern Medical Center, Dallas, United States; University of Toronto, Canada

**Keywords:** Nur77, NFATc1, osteoclast, Cbl-b, bone resorption, mouse

## Abstract

Osteoclasts are bone-resorbing cells essential for skeletal remodeling. However, over-active osteoclasts can cause bone-degenerative disorders. Therefore, the level of NFATc1, the master transcription factor of osteoclast, must be tightly controlled. Although the activation and amplification of NFATc1 have been extensively studied, how NFATc1 signaling is eventually resolved is unclear. Here, we uncover a novel and critical role of the orphan nuclear receptor Nur77 in mediating an NFATc1 self-limiting regulatory loop to prevent excessive osteoclastogenesis. Nur77 deletion leads to low bone mass owing to augmented osteoclast differentiation and bone resorption. Mechanistically, NFATc1 induces Nur77 expression at late stage of osteoclast differentiation; in turn, Nur77 transcriptionally up-regulates E3 ubiquitin ligase Cbl-b, which triggers NFATc1 protein degradation. These findings not only identify Nur77 as a key player in osteoprotection and a new therapeutic target for bone diseases, but also elucidate a previously unrecognized NFATc1→Nur77→Cblb—•NFATc1 feedback mechanism that confers NFATc1 signaling autoresolution.

**DOI:** http://dx.doi.org/10.7554/eLife.07217.001

## eLife digest

Bones are constantly remodeled in response to the stresses of everyday life. Cells called osteoclasts break down old or damaged bone and cells called osteoblasts make new bone. In healthy bones, the work of these two types of cells is well balanced. But in bone-weakening diseases like osteoporosis and certain bone cancers this balance is disturbed and the osteoclasts become overly active, leading to weak and thin bones.

Some drugs can help block the development of osteoclasts and help reduce bone loss in these diseases, but they may cause unwanted side effects. A better understanding of the processes that maintain a healthy balance of osteoblasts and osteoclasts could help scientists develop better treatments with fewer side effects. Scientists have already learned that a protein called NFATc1 turns on the production of osteoclasts. But no one knew how NFATc1 is turned off in healthy bone to prevent the excessive growth of osteoclasts and too much bone turnover.

Now, Li et al. have identified a protein called Nur77 as an important regulator of NFATc1 by examining genetically engineered mice that lack Nur77. These modified mice had more osteoclasts and thinner bones than normal mice. Further experiments used radiation to wipe out the bone marrow of normal mice, who then received bone marrow transplants from mice that lacked Nur77. After the transplant, the normal mice showed bone loss. When the experiment was reversed, and Nur77-lacking mice received bone marrow from normal mice, their bone loss was alleviated. This indicates that Nur77 acts in the bone marrow cells to control osteoclasts and skeletal health.

Li et al. found that Nur77 cannot control the expression of the gene that encodes NFATc1 or directly bind to the NFATc1 protein. Instead, Nur77 increases the production of an enzyme that breaks down the NFATc1 protein. Unexpectedly, the experiments also found that NFATc1 turns on the expression of Nur77. This means that NFATc1 essentially regulates itself by increasing its own breakdown when NFATc1 levels increase. This helps to explain how osteoclast production is normally kept in check, and may suggest new strategies for treating bone diseases.

**DOI:** http://dx.doi.org/10.7554/eLife.07217.002

## Introduction

Bone is a dynamic organ that replaces itself every 10 years in humans (Office of the Surgeon General (US), 2004). It needs to maintain an optimal density to carry out important support, movement, and protective functions in organisms. The density of bone is tightly regulated by the bone-forming osteoblasts and the bone-resorbing osteoclasts. Osteoblasts come from the mesenchymal precursor cells, whereas osteoclast precursors come from monocyte–macrophage lineage cells. Upon RANKL signaling, osteoclast precursor cells fuse and become multi-nucleated giant cells that can degrade both the organic and inorganic tissues of the bone. Osteoclast dysregulation has been associated with several human skeletal diseases such as osteoporosis, rheumatoid arthritis, and cancer metastasis to the bone (Novack and Teitelbaum, 2008). Drugs that can inhibit osteoclast differentiation, activity, or survival have been shown to be effective against these diseases (Drake et al., 2008; Body et al., 2010).

NFATc1 is a key transcriptional switch that activates osteoclastogenesis. Ectopic NFATc1 expression alone in osteoclast precursors is sufficient to produce mature osteoclasts, whereas NFATc1 deletion blocks the ability of the precursors to differentiate into osteoclasts (Takayanagi et al., 2002). NFATc1 binds to its response elements containing a consensus sequence of GGAAA, and its target genes in osteoclasts include cathepsin K, CLC-7 chloride channel, vacuolar proton pump subunit Atp6v0d2, etc (Kuroda and Matsuo, 2012). NFATc1 has been shown to auto-amplify during osteoclast differentiation, and this auto-amplification process has been suggested to be important for osteoclast lineage commitment (Asagiri et al., 2005). However, so far very few studies have dealt with whether and how NFATc1 signaling is attenuated upon its initial activation. As a result, despite the crucial functions of NFATc1 in osteoclastogenesis, the mechanisms for how NFATc1 signaling is resolved to prevent excessive osteoclast differentiation are still incompletely understood.

Nur77 (encoded by *Nr4a1*), also known as nerve growth factor IB (NGFIB), TR3 or NAK-1, is an orphan nuclear receptor in the Nr4a family, which also includes Nurr1 (*Nr4a2*) and Nor-1 (*Nr4a3*). Unlike other nuclear receptors whose functions are mainly modulated by their ligands, Nur77 is mostly regulated at the transcriptional and post-transcriptional levels (Zhao and Bruemmer, 2010). Nur77 can bind to NurRE or NBRE as monomer, homodimer, or heterodimer (Philips et al., 1997). Nur77 has been implicated in a variety of physiological processes, including thymocyte negative selection, hypothalamic-pituitary adrenal axis, chronic inflammation, and vascular smooth muscle cell proliferation (Hsu et al., 2004). Nonetheless, it is unknown whether Nur77 can directly regulate skeletal homeostasis or bone cell differentiation. In this study, we uncover a novel and important role of Nur77 in NFATc1 protein degradation during osteoclastogenesis and bone resorption, thus revealing a previously unrecognized mechanism that is essential for the resolution of NFATc1 signaling in which NFATc1 exerts self-limitation via an NFATc1→Nur77→Cblb—•NFATc1 negative feedback loop.

## Results

### Nur77 deletion enhances osteoclast differentiation

**Figure 1.**
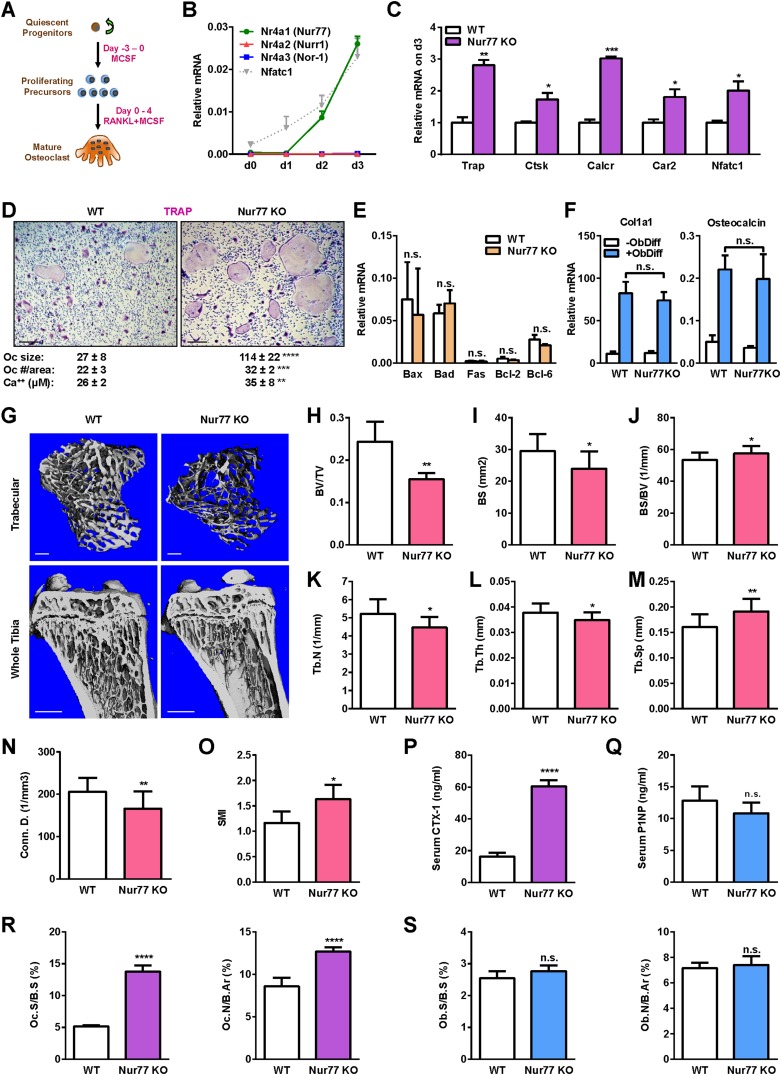
Nur77 deletion increases osteoclastogenesis and bone resorption. (**A**) A schematic diagram of *ex vivo* bone marrow osteoclast differentiation. (**B**) Expression of Nur77, Nurr1, and Nor-1 encoding genes (*Nr4a1*, *Nr4a1*, *Nr4a3*) during a time course of RANKL-induced osteoclast differentiation (n = 3 mice). *Nfatc1* mRNA level was similar to *Nr4a1* mRNA level on day 2–3. (**C**–**D**) Osteoclast differentiation was enhanced in Nur77 KO cultures compared to WT control cultures. (**C**) Expression of osteoclast differentiation markers on day 3 (n = 4 mice). (**D**) Representative images of the TRAP-stained osteoclast differentiation cultures. Mature osteoclasts were identified as multinucleated TRAP^+^ (purple) cells on day 9. Scale bar, 25 μm. Quantification of osteoclast size, osteoclast number per area, and osteoclast-resorptive activity by calcium release from bone plate to culture medium is shown (n = 4 mice in triplicate cultures). Oc, osteoclast. (**E**) Osteoclast apoptosis was unaltered, quantified by the expression of apoptosis genes on day 9 of osteoclast differentiation cultures (n = 4). (**F**) Osteoblast differentiation was unaltered in Nur77 KO cultures, measured by the expression of osteoblast markers (n = 4). (**G**–**O**) Nur77 KO mice exhibited bone loss. Tibiae from Nur77 KO mice or WT littermate controls (3 month old, male, n = 6) were analyzed by μCT. (**G**) Representative images of the trabecular bone of the tibial metaphysis (top) (scale bar, 10 μm) and the entire proximal tibia (bottom) (scale bar, 1 mm). (**H**–**O**) Quantification of trabecular bone volume and architecture. (**H**) BV/TV, bone volume/tissue volume ratio. (**I**) BS, bone surface. (**J**) BS/BV, bone surface/bone volume ratio. (**K**) Tb.N, trabecular number. (**L**) Tb.Th, trabecular thickness. (**M**) Tb.Sp, trabecular separation. (**N**) Conn.D., connectivity density. (**O**) SMI, structure model index. (**P**) Serum CTX-1 bone resorption marker was increased (3 month old, male, n = 6). (**Q**) Serum P1NP bone formation marker was unaltered (3 month old, male, n = 6). (**R**–**S**) Bone histomorphometry (3-month-old, male, n = 6). (**R**) Quantification of osteoclast surface (Oc.S/B.S) and osteoclast number (Oc.N/B.Ar). (**S**) Quantification of osteoblast surface (Ob.S/B.S) and osteoblast number (Ob.N/B.Ar). B.S, bone surface; B.Ar, bone area. Error bars, SD. **DOI:** http://dx.doi.org/10.7554/eLife.07217.003

To examine the expression pattern of Nur77 during osteoclast differentiation, we treated bone marrow-derived osteoclast precursor cells with RANKL for 4 days (Figure 1A). A time course analysis showed that *Nr4a1* mRNA started to rise on day 2 during osteoclastogenesis (Figure 1B). The other two members of the NR4A family, *Nr4a2* and *Nr4a3*, were either not expressed or expressed at much lower levels (Figure 1B). To determine if there is any potential regulatory role of Nur77 in osteoclastogenesis, we compared osteoclast differentiation cultures from bone marrow hematopoietic progenitors of Nur77 knockout (Nur77 KO) mice and WT littermate controls. The results revealed an enhanced osteoclast differentiation in Nur77 KO cultures shown by the higher expression of osteoclast differentiation markers such as tartrate-resistant acid phosphatase (*Trap*), cathepsin K (*Ctsk*), calcitonin receptor (*Calcr*), carbonic anhydrase 2 (*Car2*), as well as the master osteoclastogenic transcription factor *Nfatc1* (Figure 1C). Consistent with these results, we have also observed more and larger mature osteoclasts in the differentiation cultures (Figure 1D), as well as higher resorptive activity (Figure 1D). The expression of pro- and anti-apoptotic genes was comparable, indicating an unaffected osteoclast apoptosis (Figure 1E). In contrast, osteoblast differentiation from Nur77 KO bone marrow mesenchymal progenitors was unaltered, shown by the similar induction of osteoblast markers such as collagen, type I, alpha 1 (*Col1a1*), and *Osteocalcin* (Figure 1F). These results suggest that Nur77 may specifically suppress osteoclastogenesis during bone remodeling.

### Nur77 deletion leads to bone loss due to excessive bone resorption

To determine if Nur77 is a physiologically significant regulator of bone, we next examined the in vivo skeletal phenotype of Nur77 KO mice. MicroCT analysis revealed that male Nur77 KO mice had a low bone mass compared to WT male littermate controls (Figure 1G), illustrated by a 36% lower bone volume/tissue volume ratio (BV/TV) (Figure 1H), 19% less bone surface (BS) (Figure 1I), 8% greater bone volume/bone surface ratio (BV/BS) (Figure 1J), 14% less trabecular number (Tb.N) (Figure 1K), 8% less trabecular thickness (Tb.Th) (Figure 1L), and 19% more trabecular separation (Tb.Sp) (Figure 1M). This resulted in a 19% decrease in connectivity density (Conn.D.) (Figure 1N) and a 40% increase in the Structure Model Index (Wei et al., 2014), which quantifies the 3D structure for the relative amount of plates (SMI = 0, strong bone) and rods (SMI = 3, fragile bone) (Figure 1O). Cortical thickness was reduced by 7% (WT = 0.1125 ± 0.003 mm; KO = 0.1048 ± 0.004 mm; p = 0.02; n = 6) whereas tibia length was unaltered. Female Nur77 KO mice showed a similar phenotype with a 19% lower trabecular BV/TV (WT = 0.2025 ± 0.03; KO = 0.1632 ± 0.02; p = 0.03) and a 5% decreased cortical thickness (WT = 0.108 ± 0.003 mm; KO = 0.102 ± 0.001 mm; p = 0.04) (3 month old, n = 6).

ELISA analyses showed that the serum bone resorption marker C-terminal telopeptide fragments of the type I collagen (CTX-1) was 3.7-fold higher in Nur77 KO mice (Figure 1P), whereas the serum bone formation marker N-terminal propeptide of type I procollagen (P1NP) was unchanged (Figure 1Q). Consistent with these observations, histomorphometry of the femur showed that osteoclast surface and osteoclast number were significantly increased in Nur77 KO mice (Figure 1R), whereas osteoblast surface and osteoblast number (Figure 1S) were unaltered. Together, these results suggest that Nur77 deletion causes low bone mass primarily through increasing osteoclastogenesis and bone resorption.

### Nur77 regulation of bone resorption is intrinsic to the Osteoclast lineage

**Figure 2.**
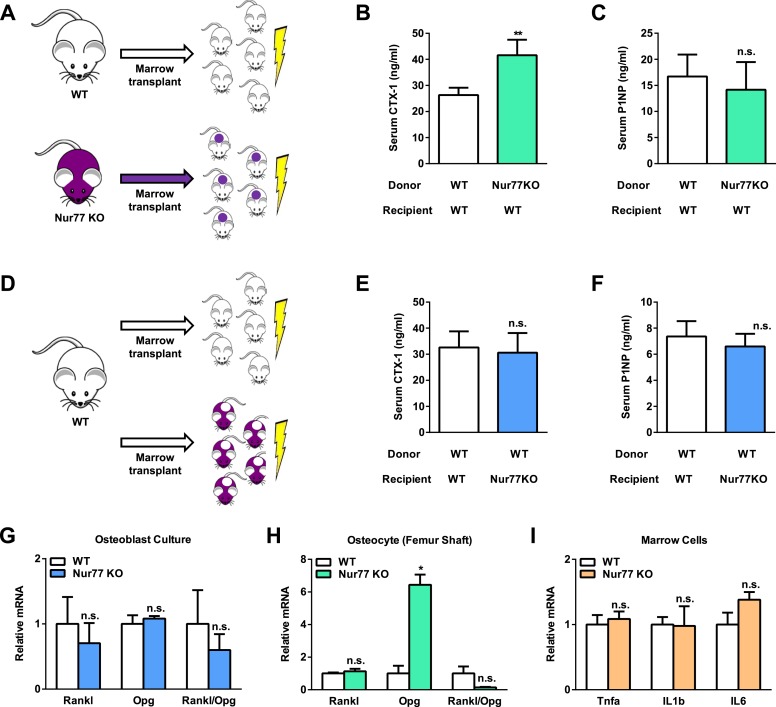
Nur77 regulation of bone resorption is intrinsic to the hematopoietic lineage. (**A**–**F**) Bone marrow transplantation. Bone marrow cells from 2-month-old male donor mice were transplanted into 2-month-old irradiated male recipient male mice (n = 5) and analyzed 3 months later at 5 month old. (**A**–**C**) Transplantation of Nur77 KO donor bone marrow cells into WT recipients conferred elevated bone resorption compared to WT control donor bone marrow cells. (**A**) A schematic diagram. (**B**) Serum CTX-1. (**C**) Serum P1NP. (**D**–**F**) Transplantation of WT donor bone marrow cells into Nur77 KO recipients rescued the bone resorption to a level similar to the WT control recipients. (**D**) A schematic diagram. (**E**) Serum CTX-1. (**F**) Serum P1NP. (**G**) Expression of RANKL and OPG, as well as RANKL/OPG ratio, in Nur77 KO *ex vivo* osteoblast differentiation cultures compared to WT control cultures (n = 3). (**H**) Expression of *Rankl* and *Opg*, as well as *Rankl*/*Opg* ratio, in osteocytes from femur shaft in Nur77 KO mice compared to WT control mice (n = 3). (**I**) Expression of pro-osteoclastogenic cytokines in bone marrow cells from Nur77 KO mice compared to WT control mice (n = 3). Error bars, SD. **DOI:** http://dx.doi.org/10.7554/eLife.07217.004

Because Nur77 has been implicated to regulate many other cell types (Hsu et al., 2004), we next performed bone marrow transplantation experiments to examine whether Nur77 regulation of bone resorption stems from the intrinsic effects in the hematopoietic/osteoclast lineage or non-autonomous effects from other tissues or cell types such as osteoblasts, osteocytes, or the neuroendocrine system. Two complimentary sets of bone marrow transplantation were performed, and serum bone markers were assessed two months later. In the first set, we harvested donor bone marrow cells from both WT and Nur77 KO mice, and transplanted them to irradiated WT recipient mice (Figure 2A). The results showed that WT mice receiving Nur77 KO bone marrow cells exhibited significantly higher CTX-1 levels than the control group (Figure 2B), but unaltered P1NP levels (Figure 2C), suggesting that Nur77 KO hematopoietic lineage was sufficient to elevate bone resorption. In the second set, we transplanted WT donor bone marrow cells into either Nur77 KO or WT control recipient mice (Figure 2D). Nur77 KO mice receiving WT bone marrow cells showed normalized CTX-1 levels similar to the control group (Figure 2E), with also similar P1NP levels (Figure 2F), suggesting that WT bone marrow can completely rescue the osteoclast defects in the Nur77 KO mice and thus other tissues/cell types play only a minor role if any. The results from these two experiments indicate that Nur77 regulation of bone resorption is intrinsic to the hematopoetic lineage.

In the bone milieu, osteoblasts and osteocytes provide RANKL and the RANKL decoy receptor OPG to stimulate and inhibit osteoclast differentiation, respectively (Boyce and Xing, 2007). Thus, we assessed whether RANKL and OPG levels were different between Nur77 KO and WT control mice. We first compared osteoblast differentiation cultures derived from Nur77 KO or WT control mice and found that there was no difference in the expression of *Rankl* or *Opg*, resulting in a comparable *Rankl*/*Opg* ratio (Figure 2G). Recently osteocytes, the mature long living osteoblasts embedded in the bone matrix, have been shown to provide the majority of RANKL and OPG to osteoclasts (Nakashima et al., 2011; Xiong et al., 2011). Hence, we compared *Rankl* and *Opg* expression in femur shafts that contain mainly osteocytes. Our results showed that there was no difference in *Rankl* expression (Figure 2H); however, Nur77 KO osteocytes express a markedly higher level of *Opg* (Figure 2H), leading to a significantly lower *Rankl*/*Opg* ratio (Figure 2H). The lower *Rankl*/*Opg* ratio in the Nur77 KO mice is unlikely to be the cause of the augmented osteoclastogenesis, but presumably an attempt of osteocytes to suppress the osteoclast over-activation. These results, coupled with unaltered in vitro osteoblast differentiation (Figure 1F) and in vivo bone formation (Figure 1Q,S), suggest that osteoblast/osteocyte are not major contributors to the excessive osteoclastogenesis and bone resorption in Nur77 KO mice.

Inflammatory cytokines in the bone microenvironment can also promote osteoclast differentiation (Zupan et al., 2013). Thus, we collected bone marrow cells from mouse femurs for gene expression assessment. No significant difference was found in the expression of *Tnfα*, *IL1β*, or *IL6* (Figure 2I). Moreover, it has been shown that serum levels of TNFa, IL1, and IL6 were unchanged in mice injected with either Nur77-expressing lentivirus or Nur77 siRNA (Hu et al., 2014), further suggesting that the augmented osteoclastogenesis in Nur77 KO mice is not due to differential levels of these inflammatory cytokines.

The above findings, together with the fact that Nur77 KO osteoclast precursors exhibited enhanced osteoclast differentiation independent of the bone and neuroendocrine environment (Figure 1C–D), indicate that Nur77 regulation of osteoclastogenesis is mainly intrinsic and cell-autonomous to the osteoclast lineage.

### Nur77 promotes NFATc1 degradation

**Figure 3.**
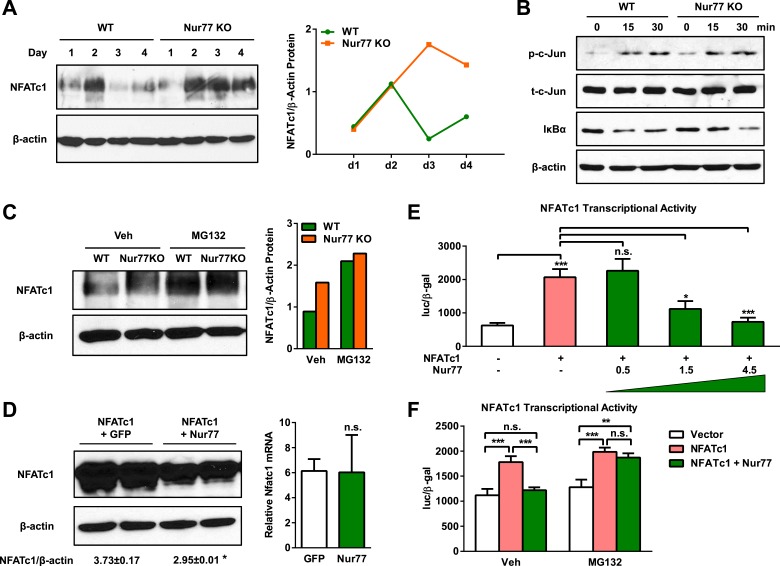
Nur77 inhibits osteoclast differentiation by promoting NFATc1 degradation. (**A**) NFATc1 protein levels during a time course of osteoclast differentiation from the bone marrow cells of Nur77 KO mice or WT control mice. Left, representative western blot image. Right, quantification of NFATc1/β-actin ratio. (**B**) c-Jun phosphorylation and IκBα degradation post RANKL treatment in osteoclast differentiation cultures from the bone marrow cells of Nur77 KO mice or WT control mice. P-c-Jun, phosphorylated c-Jun; t-c-Jun, total-c-Jun. (**C**) Effects of MG132 on NFATc1 protein levels in Nur77 KO or WT bone marrow osteoclast differentiation cultures. Cells were treated with 25 μM MG132 for 6 hr 3 days after RANKL stimulation. Left, representative western blot image. Right, quantification of NFATc1/β-actin ratio. (**D**) Effects of Nur77 over-expression on NFATc1 protein and mRNA levels. HEK293 cells were transfected with NFATc1, together with either Flag-Nur77 or GFP control. Left, representative western blot image with quantification of NFATc1/β-actin ratio. Right, relative Nfatc1 mRNA. (**E**) Effects of Nur77 over-expression on NFATc1 transcriptional output (n = 3). HEK293 cells were transfected with NFATc1 and its luciferase reporter, together with increasing amount of Nur77. (**F**) MG132 abolished the effects of Nur77 over-expression on NFATc1 transcriptional output (n = 3). HEK293 cells were treated with MG132 (25 μM) 1 day after transfection for 6 hr before harvesting. All data are representative of at least three experiments. Error bars, SD. **DOI:** http://dx.doi.org/10.7554/eLife.07217.005

Given that Nur77 exerts functions within the osteoclast itself, we decided to investigate whether Nur77 affects RANKL signaling pathways. RANKL binding to RANK receptor on osteoclast precursor cells activates AP1 transcription factor via c-Jun phosphorylation, as well as NFκB transcription factor via IκBα degradation, which in turn induces and initiates the autoamplification of NFATc1, the master transcriptional switch of osteoclastogenesis (Kuroda and Matsuo, 2012). Although *Nfatc1* mRNA level was 2-fold higher in Nur77 KO osteoclast differentiation cultures on day 3 (Figure 1C), we found that NFATc1 protein level was sevenfold higher in Nur77 KO cultures compared to WT control cultures on day 3 (Figure 3A). It has been reported that NFATc1 protein is degraded on day 3–4 during osteoclast differentiation, despite the continuously rising *Nfatc1* mRNA levels (Kim et al., 2010). Indeed, our time course analysis showed that in WT cultures, NFATc1 protein was elevated on day 2 but then rapidly down-regulated on day 3 and day 4 (Figure 3A). In contrast, in Nur77 KO cultures, the initial increase of NFATc1 protein was sustained and NFATc1 protein remained high on day 3 and day 4 (Figure 3A). We then compared c-Jun phosphorylation and IκBα degradation in the osteoclast differentiation cultures upon RANKL stimulation, but did not observe any significant difference between Nur77 KO and WT control cultures (Figure 3B), which is in agreement to the similar NFATc1 protein induction on day 1 and day 2 (Figure 3A). Moreover, there is no Nur77 binding sequence in a 4-Kb region of NFATc1 promoter. These results indicate that Nur77 regulation of NFATc1 protein level resides downstream of transcription.

It has been shown that the decrease in NFATc1 protein levels at later stage of osteoclast differentiation is due to ubiquitin-mediated protein degradation; and that MG132, a proteasome inhibitor, can restore NFATc1 protein to a similar level on day 2 (Kim et al., 2010). To examine whether the differences in NFATc1 levels between Nur77 KO and WT mice were due to protein degradation, we treated osteoclast differentiation cultures with MG132 on day 3. As our result shows, MG132 treatment increased NFATc1 protein level in WT cultures to a level similar to Nur77 KO cultures; and MG132 treatment could no longer further increase NFATc1 protein level in the Nur77 KO cultures (Figure 3C). In line with these observations, our co-IP experiments reveal that Nur77 (either endogenous or flag-tagged) does not directly interact with NFATc1 (not shown), suggesting that Nur77 does not directly modulate NFATc1's localization or activity. These results indicate that Nur77 deletion elevates NFATc1 protein levels by suppressing ubiquitin degradation pathway.

As a complementary gain-of-function approach, we tested whether Nur77 over-expression could promote NFATc1 protein degradation. We transfected HEK293 cells with NFATc1 together with Nur77 or a GFP control, and then quantified *Nfatc1* mRNA and protein levels. The result shows that Nur77 over-expression significantly decreased NFATc1 protein levels (Figure 3D, left) without altering *Nfatc1* mRNA levels (Figure 3D, right). Consistent with the lower Nur77 protein abundance, Nur77 over-expression also dosage-dependently reduced the NFATc1 transcriptional output from a luciferase reporter driven by NFATc1 response elements (Figure 3E). This Nur77 reduction of NFATc1 activity was completely abolished by MG132 (Figure 3F), indicating that it was mediated by ubiquitin degradation pathway. The mRNA expression of *Nr4a1* and *Nfatc1* in osteoclasts is comparable on day 2–3 (Figure 1B), supporting that Nur77 regulation of NFATc1 is relevant in the osteoclast. These findings further support the notion that Nur77 promotes NFATc1 protein degradation.

### Nur77 transcriptionally up-regulates E3 ligase Cbl-b

**Figure 4.**
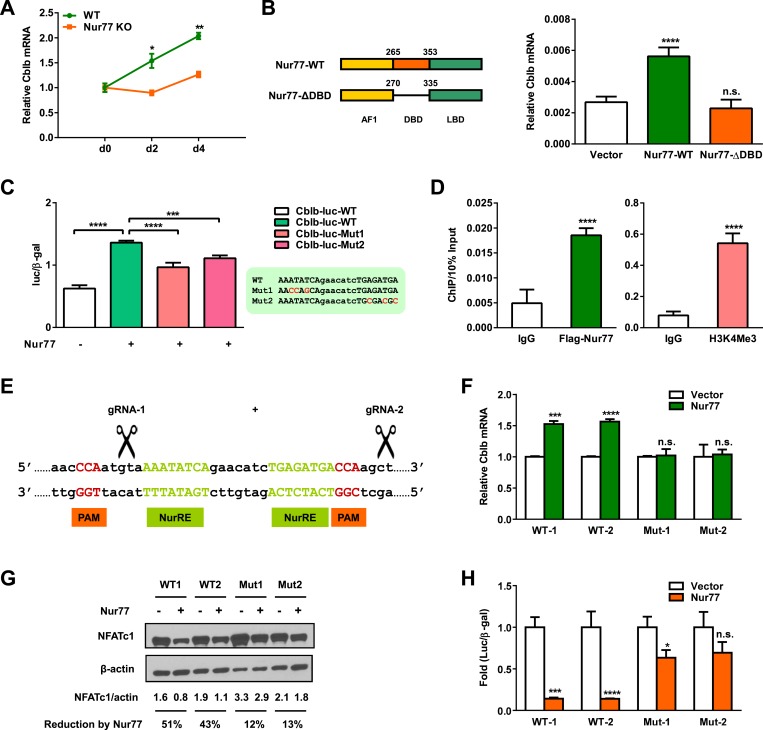
Nur77 transcriptionally up-regulates E3 ligase Cbl-b. (**A**) *Cblb* expression during a time course of osteoclast differentiation from the bone marrow cells of Nur77 KO mice or WT control mice (n = 3). (**B**) Nur77 over-expression increased *Cblb* mRNA in a DNA-binding-dependent manner. HEK293 cells were transfected with vector control, WT Nur77, or a mutant Nur77 with a deletion of the DNA binding domain (DBD) (n = 3). (**C**) Nur77 activated Cbl-b promoter via NurRE. HEK293 cells were transfected with Nur77, together with a luciferase vector control or a luciferase reporter driven by 1 Kb Cbl-b promoter containing either a WT NurRE or a mutant NurRE (n = 3). Inset shows the mutations in the two mutant reporters. (**D**) ChIP assay of Nur77 binding and H3K4me3 levels at the endogenous Cbl-b promoter. HEK293 cells were transfected with Flag-Nur77, Nur77 binding were detected with anti-Flag antibody and compared with IgG control antibody (n = 3). (**E**–**H**) CRISPR/Cas9 deletion of NurRE in the endogenous Cbl-b promoter abolished Nur77 regulation of Cbl-b and NFATc1. (**E**) A schematic representation of CRISPR/Cas9 gRNAs and their target locus in the Cbl-b promoter. (**F**–**H**) Effects of Nur77 over-expression on Cbl-b mRNA (**F**) (n = 4), NFATc1 protein (**G**), and NFATc1 transcriptional output (**H**) (n = 3) in two independent HEK293 CRISPR mutant clones and WT controls. Error bars, SD. **DOI:** http://dx.doi.org/10.7554/eLife.07217.006

In the ubiquitin degradation pathway, E3 ligases are responsible for substrate specificity and ubiquitination regulation. We next searched for E3 ligases that could be responsible for NFATc1 degradation in osteoclasts. It has been reported that Cbl-b, an E3 ligase in the Cbl family, is a major contributor to the ubiquitin-mediated down-regulation of NFATc1 at late stage of osteoclast differentiation (Kim et al., 2010). Therefore, we tested the hypothesis that Nur77 may promote NFATc1 degradation by inducing Cbl-b. We found that *Cblb* expression was significantly lower in Nur77 KO osteoclast differentiation cultures compared to WT control cultures on day 2 and day 4 (Figure 4A). Conversely, Nur77 over-expression in HEK293 cells significantly increased *Cblb* expression (Figure 4B). Importantly, a truncated Nur77 mutant in which the DNA binding domain (DBD) was deleted could no longer up-regulate *Cblb*, suggesting that Nur77 induction of *Cblb* transcription depends on its DNA binding ability (Figure 4B). The functional connection between Nur77 and Cbl-b is further supported by the similar bone phenotype in Nur77 KO mice (Figure 1) and Cblb KO mice (Nakajima et al., 2009), including increased *ex vivo* osteoclast differentiation and in vivo bone resorption, but unaltered bone formation, leading to lower bone mass.

To investigate whether *Cblb* is a direct Nur77 target gene, we tested whether Nur77 could transcriptionally activate the Cbl-b promoter. We cloned a 1-kb segment of Cbl-b promoter upstream of a luciferase reporter and tested its expression in a transient transfection assay in HEK293 cells. The result showed that co-transfection with Nur77 significantly up-regulated the Cbl-b promoter activity by 2.2-fold (Figure 4C), suggesting that Nur77 is able to directly activate *Cblb* transcription. Bioinformatic analyses revealed a pair of motifs that may comprise a Nur77 response element (NurRE) in the Cbl-b promoter at ∼600 bp upstream of the transcription start site (Figure 4C, inset). To examine whether these putative NurRE motifs are important for Nur77 induction of Cbl-b promoter, we mutated each NurRE motif to derive mutant-1 and mutant-2 luciferase reporters (Figure 4C, inset). Both mutant reporters exhibited a significantly compromised ability to be activated by Nur77 compared to the WT reporter (Figure 4C), indicating that both NurRE motifs are functionally required. To determine whether Nur77 can bind to the endogenous Cbl-b NurRE, we performed chromatin immunoprecipitation (ChIP) assay. Nur77 was found to be enriched at the NurRE region, leading to transcription activation shown by the presence of H3K4Me3 histone mark at the transcription start site (Figure 4D). These results suggest that Nur77 can directly induce Cbl-b transcription by binding to a NurRE in the Cbl-b promoter.

We next sought to elucidate whether Nur77 induction of Cbl-b is functionally required for Nur77 down-regulation of NFATc1 protein. Instead of deleting *Cblb*, we designed a more prudent strategy to specifically disrupt the NurRE region in the endogenous Cbl-b promoter using CRISPR/Cas9 genome editing tool, thus more precisely dissecting the functional interaction among Nur77, Cbl-b, and NFATc1 (Figure 4E). Compared with WT control cells, the ability of Nur77 to increase *Cblb* mRNA (Figure 4F), as well as to decrease NFATc1 protein level (Figure 4G) and transcriptional output (Figure 4H), was significantly attenuated in two independent CRISPR mutant clones. These results provide strong evidence that Nur77 promotes NFATc1 protein degradation by directly inducing the transcription of Cbl-b E3 ligase.

### NFATc1 up-regulates Nur77 to form a self-limiting loop

**Figure 5.**
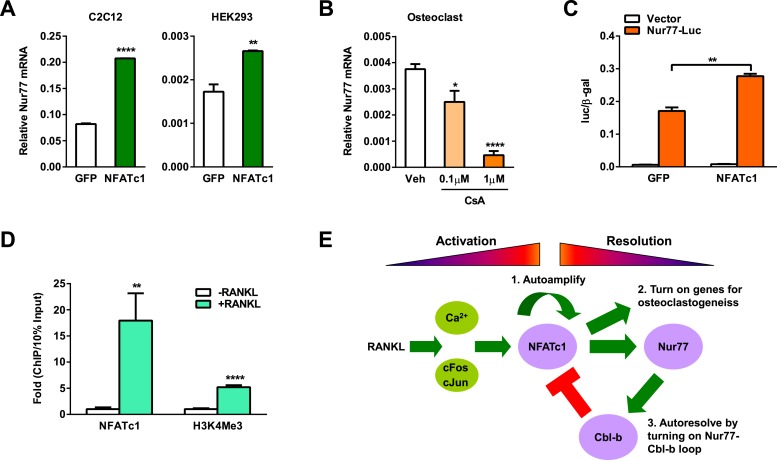
NFATc1 induces Nur77 transcription to elicit a self-limiting loop. (**A**) NFATc1 over-expression increased Nur77 mRNA. Mouse myoblast cell line C2C12 or human embryonic kidney cell line HEK293 were transfected with NFATc1 or GFP control. (**B**) NFATc1 inhibition by Cyclosporin A dosage-dependently decreased Nur77 mRNA. Osteoclast differentiation cultures were treated with Cyclosporin A on day 2 for 24 hr (n = 4). (**C**) NFATc1 over-expression enhances Nur77 promoter activity. HEK293 cells were transfected with a luciferase vector control or a luciferase reporter driven by a 0.8 Kb Nur77 promoter, together with NFATc1 or GFP control (n = 3). (**D**) ChIP assay of NFATc1 binding and H3K4me3 level at the endogenous Nur77 promoter in RAW264.7 mouse macrophage cell line with or without 2 day RANKL stimulation. (**E**) A working model of an NFATc1 self-limiting loop in which NFATc1 elicits its own degradation by inducing Nur77 and consequently Cbl-b to resolve NFATc1 signaling. Error bars, SD. **DOI:** http://dx.doi.org/10.7554/eLife.07217.007

Since Nur77 expression consistently rises during osteoclastogenesis (Figure 1B), we hypothesize that there is an upstream regulator that induces Nur77 transcription upon RANKL signaling activation. Interestingly, we found several NFATc1 response elements in the Nur77 promoter region, suggesting that NFATc1 up-regulates Nur77 to initiate a negative feedback loop. To test whether NFATc1 itself is sufficient to increase Nur77 expression independent of other RANKL signaling pathways, we performed transfection assays to over-express NFATc1. Compared to a GFP negative control, NFATc1 over-expression significantly increased Nur77 expression in both HEK293 cells and mouse myoblast C2C12 cells (Figure 5A). Conversely, treatment of osteoclast differentiation cultures with cyclosporin A, a calcineurin inhibitor that suppresses NFATc1 activity, dosage-dependently decreased Nur77 expression (Figure 5B).

We next examined whether NFATc1 could directly activate Nur77 promoter. We cloned a 0.8 Kb Nur77 promoter region upstream of a luciferase reporter and tested its inducibility by NFATc1 by transient transfection. Compared to a GFP negative control, NFATc1 over-expression significantly elevated the luciferase output (Figure 5C). Moreover, ChIP assay showed that RANKL treatment of osteoclast differentiation cultures markedly increased NFATc1 binding to the endogenous Nur77 promoter (Figure 5D), leading to activated Nur77 transcription as shown by the higher level of H3K4Me3 histone mark at the transcription start site (Figure 5D). Together, these results indicate that Nur77 is a direct transcriptional target of NFATc1, and thus revealing a key mechanism for how NFATc1 resolves its own signaling to prevent excessive osteoclastogenesis via an NFATc1→Nur77→Cblb—•NFATc1 self-limiting loop (Figure 5E).

### Cbl-b deletion abolishes Nur77 regulation of osteoclastogenesis and bone resorption

**Figure 6.**
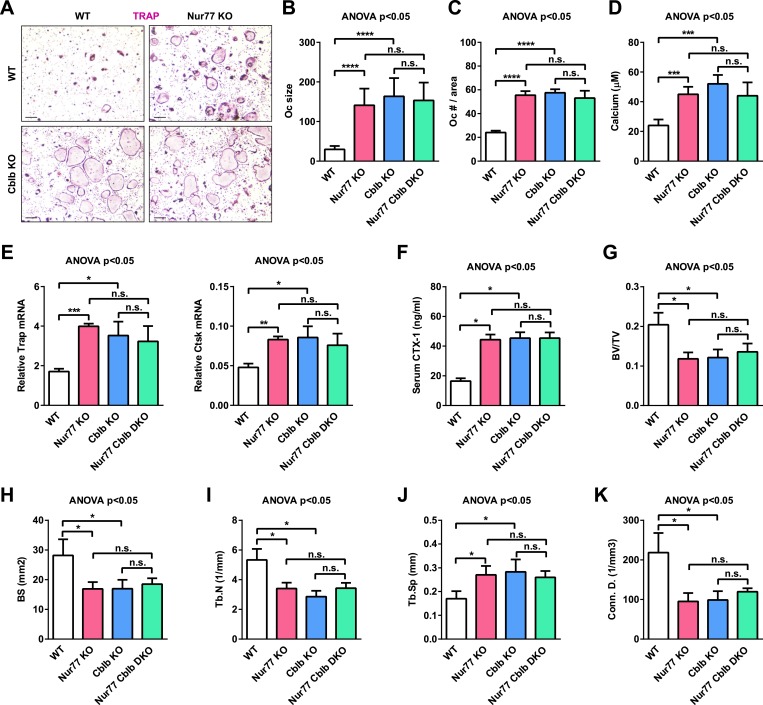
Cbl-b deletion abolishes Nur77 regulation of osteoclastogenesis and bone resorption. Nur77 Cblb DKO mice were compared with Nur77 KO mice, Cblb KO mice, and WT littermate control mice (3 month old, male, n = 4). (**A**–**E**) *Ex vivo* bone marrow osteoclast differentiation (n = 4 mice in triplicate cultures). (**A**) Representative images of the TRAP-stained osteoclast differentiation cultures. Mature osteoclasts were identified as multinucleated TRAP^+^ (purple) cells on day 9. Scale bar, 25 μm. (**B**) Osteoclast size. (**C**) Osteoclast number per area. (**D**) Osteoclast-resorptive activity by calcium release from bone plate to culture medium. (**E**) Expression of osteoclast differentiation markers on day 3. (**F**) ELISA analysis of serum CTX-1 bone resorption marker (n = 4). (**G**–**K**) μCT analysis of trabecular bone parameters (n = 4). (**G**) BV/TV, bone volume/tissue volume ratio. (**H**) BS, bone surface. (**I**) Tb.N, trabecular number. (**J**) Tb.Sp, trabecular separation. (**K**) Conn.D., connectivity density. Statistical analyses were performed with ANOVA followed by the post-hoc Tukey pairwise comparisons. Error bars, SD. **DOI:** http://dx.doi.org/10.7554/eLife.07217.008

To further determine whether Cbl-b is required for the anti-osteoclastogenic function of Nur77 in vivo, we conducted genetic experiments by comparing Nur77 Cblb double knockout mice (DKO) with Nur77 KO mice, Cblb KO mice, and WT littermate controls. *Ex vivo* bone marrow osteoclast differentiation assay showed that Nur77 deletion could no longer further enhance osteoclast differentiation in the absence of Cbl-b; furthermore, Cblb KO cultures and Nur77 Cblb DKO cultures showed a similar enhanced osteoclastogenesis as Nur77 KO cultures compared with WT control cultures (Figure 6A–E). In accordance to this *ex vivo* finding, in vivo analyses showed that when Cbl-b is absent, Nur77 deletion could no long further elevate serum bone resorption (Figure 6F) or reduce bone mass (Figure 6G–K); Cblb KO mice and Nur77 Cblb DKO mice showed a similar high bone resorption and low bone mass phenotype as Nur77 KO mice compared with WT controls (Figure 6F–K). These findings demonstrate that Cbl-b deletion fully recapitulates Nur77 deletion, and Cbl-b deletion completely abolishes Nur77 regulation of osteoclastogenesis and bone resorption. Therefore, this in vivo genetic evidence strongly supports Cbl-b as a major and essential mediator of Nur77 function in the osteoclast lineage.

## Discussion

In this study, we have identified the nuclear receptor Nur77 as a critical negative regulator of osteoclastogenesis and bone resorption, revealing its novel bone protective role. Nur77 deletion causes elevated bone resorption and bone loss in mice. Moreover, we have also unraveled a previously unrecognized mechanism for how Nur77 attenuates NFATc1 signaling at late stage of osteoclast differentiation. Nur77 transcriptionally up-regulates Cbl-b E3 ligase to trigger NFATc1 protein degradation, so that NFATc1 signaling can be resolved in a timely fashion to prevent excessive osteoclastogenesis and bone resorption (Figure 5E).

When osteoclast differentiation is enhanced in a culture, typically all RANKL-induced osteoclastogenic genes will be elevated, including *Nfatc1*, because the gene expression analysis is a population-based assay (Figure 1C). In addition to induction by RANKL-activated upstream signaling, NFATc1 also auto-amplifies itself at mRNA level (Asagiri et al., 2005), which makes it harder to discern the origin of NFATc1 regulation in osteoclast cultures. Thus, we subsequently examined the intrinsic ability of Nur77 to regulate NFATc1 via transfection in a heterologous cell type such as HEK293 cells, in the absence of the other RANKL signaling in osteoclast cultures. We show that Nur77 can decrease NFATc1 protein levels without affecting *Nfatc1* mRNA levels (Figure 3D). Also, there is no Nur77 binding sequence in NFATc1 promoter, further support that Nur77 does not directly regulate NFATc1 transcription. Moreover, our co-IP experiment show that Nur77 (either endogenous or a flag-tagged) does not directly interact with NFATc1, suggesting that Nur77 does not directly modulate NFATc1 localization or activity. As a result, we conclude that Nur77 mainly regulates NFATc1 protein degradation.

In searching for the Nur77-induced NFATc1-targeting E3 ubiquitin ligase, we have considered both of the two members in the Cbl family—Cbl and Cbl-b. Findings from our study and previous studies indicate that Cbl-b, but not Cbl, is the major regulator in osteoclast due to the following reasons: (1) previous studies and our data show that Cbl-b KO mice exhibit enhanced osteoclast differentiation, higher bone resorption and lower bone mass that is similar to the phenotype we observed in Nur77 KO mice (Nakajima et al., 2009) (Figure 6). In contrast, adult Cbl KO mice have no obvious bone phenotype (Chiusaroli et al., 2003). This supports that Cbl-b, but not Cbl, is a physiologically significant regulator of osteoclast in vivo. (2) Nur77 over-expression induces the expression of Cbl-b, but not Cbl. (3) A complete NurRE exists only in Cbl-b promoter, but not in Cbl promoter. (4) Nur77 KO osteoclast cultures show a significant lower level of Cbl-b, but not Cbl. (5) Finally, our in vivo genetic data comparing Nur77 Cblb DKO mice with Cblb KO mice show that Cbl-b deletion completely abolishes the enhanced osteoclast differentiation, elevated bone resorption, and reduced bone mass in Nur77 KO mice, demonstrating that Cbl-b is the major and essential mediator of Nur77 regulation of osteoclastogenesis, which is not compensated by Cbl.

In the process of studying the role of Nur77 in NFATc1 regulation and osteoclast differentiation, we inadvertently discovered that Nur77 is not only a regulator of NFATc1 but also a transcriptional target of NFATc1, thus revealing a negative feedback loop where NFATc1 induces its own degradation by up-regulating Nur77 and Cbl-b. This mechanism is crucial for proper cellular differentiation and function, since the resolution of signaling is just as important as its initiation and amplification. A breach in the NFATc1→Nur77→Cblb—•NFATc1 regulatory loop, exemplified by the Nur77 KO mice, will cause pathologically elevated NFATc1 levels during late stage of osteoclastogenesis and send osteoclasts into overdrive. To the best of our knowledge, we are the first group to propose an NFATc1 self-limiting regulatory mechanism. Therefore, NFATc1 exerts three functions to control osteoclastogenesis. In addition to the previously recognized roles in its auto-amplification and activating osteoclast genes to initiate the differentiation, NFATc1 also plays a role in its auto-resolution to cease the differentiation (Figure 5E). Our findings will pave the road for future investigations to examine whether this NFATc1→Nur77→Cblb—•NFATc1 negative feedback loop may be widely applicable to NFATc1 regulation of other cellular processes such as T cell activation and cancer development.

Identification of novel osteoclast signaling pathways provides insights into potential new therapeutic options to treat bone-degenerative diseases by inhibiting osteoclast activity. Current clinically approved osteoclast inhibitors, such as bisphosphonates and denosumab (anti-RANKL antibody), may cause severe side effects such as osteonecrosis of the jaw (ONJ) (Khan et al., 2009; Boquete-Castro et al., 2015). These side effects could stem from a variety of factors including target specificity. The whole body Nur77 KO mice, however, provides an example where despite the wide spread expression of a gene, it still can be targeted due to the differential sensitivity in different tissues. Although Nur77 has been implicated in numerous physiological functions in vitro, most of these functions did not hold true in vivo based on a general lack of phenotype in Nur77 KO mice (Lee et al., 1995; Chao et al., 2013). Until recently, Nur77 KO mice largely appear healthy and normal, and only exhibit clinical deficiencies under severe stress (Chao et al., 2009; Palumbo-Zerr et al., 2015) or with the deletion of an additional NR4A gene (Mullican et al., 2007). This may be partially due to functional redundancy among NR4A family members so that the compensation by Nurr1 and Nor1 masks the effects of Nur77 loss. Interestingly, we found that Nur77 is the predominant NR4A member in the osteoclast lineage with little or no Nurr1 or Nor1 expression (Figure 1B), explaining the critical role of Nur77 in osteoclastogenesis so that the effects on bone is evident by Nur77 deletion alone. This creates an exciting opportunity for selective drug targeting and precision medicine with minimal side effects. Most recently, Tontonoz et al. have uncovered a muscle protective role of Nur77 as mice deficient in Nur77 alone exhibit reduced muscle mass and myofiber size (Tontonoz et al., 2015). Therefore, Nur77 activation may represent a promising therapeutic strategy for musculoskeletal degenerative diseases with dual benefits on muscle and bone.

The crucial regulation of NFATc1 protein degradation by Nur77 and Cbl-b suggests that it may be therapeutically beneficial to accelerate RANKL signaling resolution during osteoclastogenesis. Indeed, defects in the components of ubiquitin and proteasome system have been implicated in diseases including cancer and neurodegenerative disorders (Popovic et al., 2014). Bortezomib, a peptide inhibitor of proteasome, has been approved for clinical usage in pathological settings such as refractory multiple myeloma (Richardson et al., 2005). The discovery of pathway-specific ubiquitin–proteasome activators, however, is somewhat lagging behind. Nonetheless, oleuropein, a small molecule proteasome activator, has been shown to delay replicative senescence of human embryonic fibroblast (Katsiki et al., 2007). In addition, oleuropein treatment has been shown to inhibit osteoclast formation and suppress the loss of trabecular bone in ovariectomized mice (Hagiwara et al., 2011), giving hope that small molecules that selectively activate the protein degradation pathway may be a promising future therapeutic strategy for skeletal and other diseases.

## Materials and methods

### Mice

Nur77 KO mice (Lee et al., 1995) in a C57BL/6 and 129SvJ hybrid background was originally generated by Jeffrey Milbrandt at Washington University School of Medicine and kindly provided by Orla Conneely at Baylor College of Medicine. Cblb KO mice in a mixed background were originally generated by Josef Penninger (Bachmaier et al., 2000). Mice were fed with standard chow ad libitum and kept on a 12-hr light, 12-hr dark cycle. Nur77 KO mice were bred with Cblb KO mice to generate Nur77 Cblb double heterozygous mice, which were then bred to generate littermates for Nur77 KO, Cblb KO, Nur77 Cblb DKO, and WT control. All experiments were conducted using littermates. Bone marrow transplantation was performed as described (Wan et al., 2007; Krzeszinski et al., 2014). Briefly, bone marrow cells from 2-month-old male donor (WT or Nur77 KO) were intravenously transplanted via retro orbital injection into 2-month-old male recipients (WT or Nur77 KO) that were irradiated at lethal dose (1000 roentgen); the mice were analyzed 3 month post transplantation. Our established protocol for lethal irradiation and bone marrow transplantation achieves >95% repopulation of donor cells in the recipient mice, as measured by the percentage of CD45.1 vs CD45.2. Sample size estimate was based on power analyses performed using SAS 9.3 TS X64_7PRO platform at the UTSW Biostatistics Core. With the observed group differences and the relatively small variation of the in vivo measurements, n = 4 and n = 3 will provide >90% and >80% power at type I error rate of 0.05 (two-sided test), respectively. All protocols for mouse experiments were approved under number 2008-0324 by the Institutional Animal Care and Use Committee of UTSW.

### Bone analyses

μCT was performed using a Scanco μCT-35 instrument (Scanco Medical, Brüttisellen, Switzerland) as described (Wei et al., 2010). Histomorphometry were performed as described (Wan et al., 2007; Wei et al., 2011). Serum CTX-1 bone resorption marker and P1NP bone formation marker were measured with RatLaps EIA kit and Rat/Mouse PINP EIA kit (Immunodiagnostic Systems, Tyne & Wear, United Kingdom), respectively. To analyze osteocyte gene expression, mouse femur was cut off at both ends to allow marrow cells to be flushed out with media. It was then soaked in PBS and spun down to remove residual marrow cells, and snap frozen in liquid nitrogen, stored at −80°C until RNA extraction.

### *Ex vivo* osteoclast and osteoblast differentiation

Osteoclasts were differentiated from bone marrow cells as described (Wan et al., 2007). Briefly, hematopoietic bone marrow cells were purified with a 40-μm cell strainer, cultured for 16 hr with 5 ng/ml MCSF (R&DSystems, Minneapolis, Minnesota) in α-MEM containing 10% FBS. Floating cells were then collected and differentiated with 40 ng/ml of M-CSF in α-MEM containing 10% FBS for 3 days (day −3 to day 0), then with 40 ng/ml of MCSF, and 100 ng/ml of RANKL (R&D Systems) for 3–9 days (day 0 to day 9). Mature osteoclasts were identified as multinucleated (>3 nuclei) TRAP^+^ cells on day 9. Osteoclast differentiation and apoptosis were quantified by the RNA expression of osteoclast markers and apoptosis genes on day 3 and 6, respectively, using RT-QPCR analysis. For osteoclast-resorptive function analyses, osteoclast differentiation was conducted in OsteoAssay bone plates (Lonza, Basel, Switzerland), and osteoclast activity was quantified as calcium release from bone into culture medium using a colorimetric calcium detection kit (Abcam, Cambridge, Massachusetts). Osteoblast differentiation from bone marrow cells was performed as previously described (Wei et al., 2012, 2014). Briefly, bone marrow cells were cultured for 4 days in MSC media (Mouse MesenCult Proliferation Kit, StemCell Technologies), then differentiated into osteoblast with α-MEM containing 10% FBS, 5 mM β-glycerophosphate, and 100 μg/ml ascorbic acid for 9 days.

### Reagents

Antibodies for NFATc1, total-c-Jun and IκBα, as well as cyclosporine A were purchased from Santa Cruz Biotechnology (Dallas, Texas). Phospho (ser73)-c-Jun antibody was from Cell Signaling (Beverly, Massachusetts). Anti-Histone H3 (tri methyl K4) antibody was from Abcam. Antibodies for Flag and β-actin were from Sigma (St. Louis, Missouri). MG132 was from Fisher (Pittsburgh, Pennsylvania). Western blot and ChIP assays were performed as previously described (Wan et al., 2007; Krzeszinski et al., 2014). NFATc1 expression plasmid was purchased from Open Biosystems (Lafayette, Colorado). Human Flag-Nur77 expression plasmid was kindly provided by Orla Conneely lab. Nur77-ΔDBD expression plasmid was constructed by deleting the amino acid residues 270–335 from the WT Nur77 expression plasmid. RNA was reverse transcribed into cDNA using an ABI High Capacity cDNA RT Kit (Life Technologies, Carlsbad, California) and analyzed using real-time quantitative PCR (SYBR Green) in triplicate. All RNA expression was normalized by the ribosomal gene L19.

### Promoter analyses

Cbl-b-promoter-luc-WT and Nur77-promoter-luc were constructed by cloning 1-Kb and 0.8-Kb segment upstream of transcription start site into pGL4 luciferase vector. Cbl-b-luc-Mut1 and Cbl-b-luc Mut2 were created by introducing mutations to three residues in each NurRE region of Cbl-b promoter, using the QuikChange II XL Site-Directed Mutagenesis Kit (Agilent Technologies, Santa Clara, California). NFATc1 transcriptional activity was quantified using pNFAT-Luc reporter (Agilent Technologies). For transient transfection, a luciferase reporter was co-transfected into HEK293 cells with expression plasmids for β-gal and factors to be tested using FuGENE HD reagent (Roche, Basel, Switzerland). Vector alone or a GFP expression plasmid served as a negative control. Luciferase activity was measured 48 hr later and normalized by β-gal activity. All transfection experiments were performed in triplicates and repeated for at least three times.

### CRISPR Constructs and clone Screening

Plasmids for gRNA cloning and hCas9 expression were from Addgene (Cambridge, Massachusetts). Oligos for gRNA were designed to target upstream and downstream of the NurRE in the Cbl-b promoter and cloned into gRNA vector according to the instruction form George Church Laboratory. Both vectors for gRNAs and the expression plasmids for hCas9 and GFP marker were co-transfected into HEK293 cells. GFP^+^ cells were sorted into 96-well plates at 1 cell/well 48 hr later. Each clone was expanded; genomic DNA was amplified by PCR and genotyped by sequencing. Two independent clones with NurRE deletion were compared to WT control.

### Statistical analyses

All statistical analyses were performed with Student's t-test and represented as mean ± standard deviation unless noted otherwise. For in vivo experiments with ≥3 groups, statistical analyses were performed with ANOVA followed by the post-hoc Tukey pairwise comparisons. The p values were designated as: *, p < 0.05; **, p < 0.01; ***, p < 0.005; ****, p < 0.001; n.s. non-significant (p > 0.05).
